# *N*′-(3,4-Dihy­droxy­benzyl­idene)-2-meth­oxy­benzohydrazide

**DOI:** 10.1107/S1600536812025688

**Published:** 2012-06-13

**Authors:** Tong Shen, Guoli Li, Bin Zheng

**Affiliations:** a School of Chemical and Biological Engineering, Lanzhou Jiaotong University, Lanzhou 730070, People’s Republic of China; b Engineering and Technology Center of Gansu Province for Botanical Pesticides, Lanzhou Jiaotong University, Lanzhou 730070, People’s Republic of China

## Abstract

The title compound, C_15_H_14_N_2_O_4_, was prepared from 3,4-dihy­droxy­benzaldehyde and 2-meth­oxy­benzhydrazide in absolute methanol. An intra­molecular N–H⋯O hydrogen bond makes an *S*(6) ring motif and the dihedral angle between the aromatic rings is 3.2 (3)°. The *meta*-O atom is disordered over two positions in a 0.809 (6):0.191 (6) ratio. The crystal structure features O—H⋯N and O—H⋯O hydrogen bonds.

## Related literature

For the structures and biological aspects of benzohydrazone derivatives, see: Horkaew *et al.* (2012 ▶); Rassem *et al.* (2012 ▶); Zhang *et al.* (2012 ▶); Fun *et al.* (2011 ▶). For hydrogen-bond motifs, see: Bernstein *et al.* (1995 ▶);. 
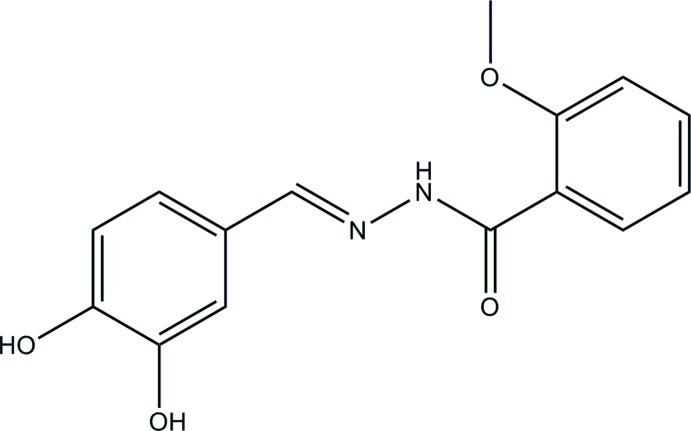


## Experimental

### 

#### Crystal data

C_15_H_14_N_2_O_4_
*M* *_r_* = 286.28
Orthorhombic, 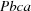

*a* = 13.796 (2) Å
*b* = 8.412 (2) Å
*c* = 24.004 (3) Å
*V* = 2785.7 (9) Å^3^
*Z* = 8
Mo *K*α radiation
μ = 0.10 mm^−1^
*T* = 298 K
0.13 × 0.10 × 0.10 mm

#### Data collection

Bruker SMART 1K CCD area-detector diffractometer
Absorption correction: multi-scan (*SADABS*; Sheldrick, 1996 ▶) *T*_min_ = 0.987, *T*_max_ = 0.990
12495 measured reflections
2570 independent reflections
1231 reflections with *I* > 2σ(*I*)
*R*_int_ = 0.093

#### Refinement

*R*[*F* ^2^ > 2σ(*F* ^2^)] = 0.068
*wR*(*F* ^2^) = 0.159
*S* = 1.03
2570 reflections
205 parameters
3 restraints
H atoms treated by a mixture of independent and constrained refinement
Δρ_max_ = 0.24 e Å^−3^
Δρ_min_ = −0.18 e Å^−3^

### 

Data collection: *SMART* (Bruker, 2007 ▶); cell refinement: *SAINT* (Bruker, 2007 ▶); data reduction: *SAINT*; program(s) used to solve structure: *SHELXS97* (Sheldrick, 2008 ▶); program(s) used to refine structure: *SHELXL97* (Sheldrick, 2008 ▶); molecular graphics: *SHELXTL* (Sheldrick, 2008 ▶); software used to prepare material for publication: *SHELXTL*.

## Supplementary Material

Crystal structure: contains datablock(s) I, global. DOI: 10.1107/S1600536812025688/hb6829sup1.cif

Structure factors: contains datablock(s) I. DOI: 10.1107/S1600536812025688/hb6829Isup2.hkl

Additional supplementary materials:  crystallographic information; 3D view; checkCIF report

## Figures and Tables

**Table 1:** Hydrogen-bond geometry (Å, °)

*D*—H⋯*A*	*D*—H	H⋯*A*	*D*⋯*A*	*D*—H⋯*A*
O4—H4⋯O2^i^	0.82	1.91	2.730 (3)	174
O3—H3*B*⋯N2^i^	0.82	2.31	2.789 (4)	118
O3—H3*B*⋯O2^i^	0.82	2.36	3.166 (4)	167
N1—H1⋯O1	0.90 (1)	1.88 (3)	2.620 (4)	138 (3)

Symmetry code: (i) 
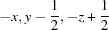
.

**Fig. 1.**
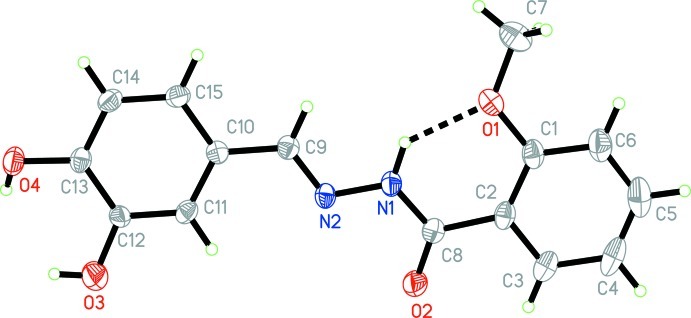
The molecular structure of the title compound, showing 30% probability displacement ellipsoids. Hydrogen bond was drawn as a dashed line. Only the major component of the disordered group is shown.

**Fig. 2.**
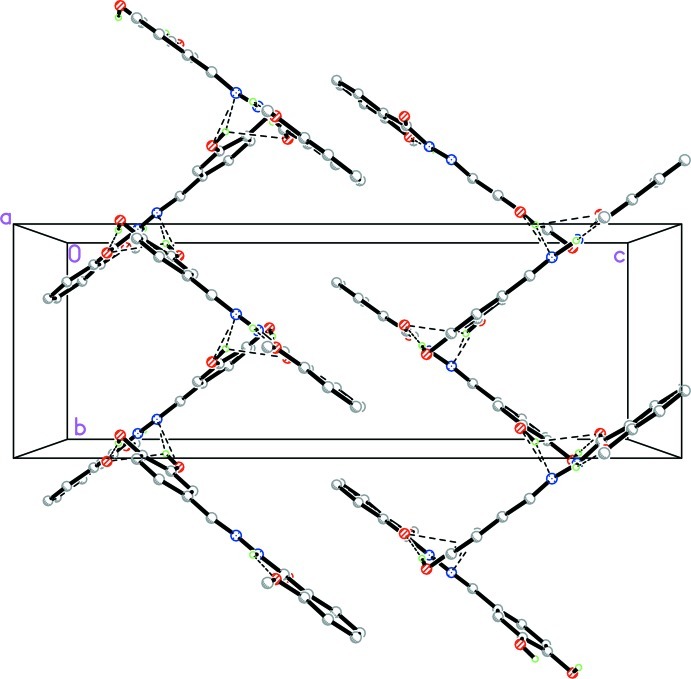
A crystal packing diagram of the title compound viewed along the a axis. Hydrogen bonds were drawn as dashed lines.

## supplementary crystallographic information

### Comment

In recent years, benzohydrazone derivatives have received much attention especially for their structures and biological aspects (Horkaew *et al.*, 2012; Rassem *et al.*, 2012; Zhang *et al.*, 2012; Fun *et al.*, 2011). We report herein the title new benzohydrazone derivative, (I).

The molecule of the title compound displays a *trans*-configuration about the C9 ═N2 bond (Fig. 1). An intramolecular N–H···O hydrogen bond makes an S(6) ring motif (Bernstein *et al.*, 1995). The dihedral angle between the aromatic rings C1—C6 and C10—C15 is 3.2 (3)°. In the crystal, molecules are linked by O–H···N, O–H···O, and N–H···O hydrogen bonds (Table 1) to form one-dimensional zigzag chains along the *b* axis (Fig. 2).

### Experimental

The title compound was prepared by stirring 3,4-dihydroxybenzaldehyde (1 mmol, 0.14 g) and 2-methoxybenzohydrazide (1 mmol, 0.17 g) in absolute methanol (30 ml). The mixture was refluxed for 1 h. The solution was then cooled to room temperature. Colorless blocks were recrystallized from methanol by slow evaporation of the solvent at room temperature after a few days.

### Refinement

The amide H atom was located in a difference map and refined isotropically [N–H = 0.90 (1) Å]. The remaining H atoms were positioned geometrically and allowed to ride on their parent atoms, with C–H = 0.93 Å for aromatic and CH and 0.96 Å for CH_3_ atoms, O–H = 0.82 Å. The *U*_iso_ values were constrained to be 1.5*U*_eq_ of the carrier atom for methyl and hydroxyl H atoms and 1.2*U*_eq_ for the remaining H atoms. The O3 atom is disordered over two sites with occupancies of 0.809 (2) and 0.191 (2), respectively.

### Crystal data

**Table:**

C_15_H_14_N_2_O_4_	*D*_x_ = 1.365 Mg m^−^^3^
*M**_r_* = 286.28	Mo *K*α radiation, λ = 0.71073 Å
Orthorhombic, *P**b**c**a*	Cell parameters from 1590 reflections
*a* = 13.796 (2) Å	θ = 2.9–26.4°
*b* = 8.412 (2) Å	µ = 0.10 mm^−^^1^
*c* = 24.004 (3) Å	*T* = 298 K
*V* = 2785.7 (9) Å^3^	Block, colorless
*Z* = 8	0.13 × 0.10 × 0.10 mm
*F*(000) = 1200	

### Data collection

**Table:**

Bruker SMART 1K CCD area-detector diffractometer	2570 independent reflections
Radiation source: fine-focus sealed tube	1231 reflections with *I* > 2σ(*I*)
Graphite monochromator	*R*_int_ = 0.093
ω scan	θ_max_ = 25.5°, θ_min_ = 3.0°
Absorption correction: multi-scan (*SADABS*; Sheldrick, 1996)	*h* = −15→16
*T*_min_ = 0.987, *T*_max_ = 0.990	*k* = −10→10
12495 measured reflections	*l* = −29→22

### Refinement

**Table:**

Refinement on *F*^2^	Primary atom site location: structure-invariant direct methods
Least-squares matrix: full	Secondary atom site location: difference Fourier map
*R*[*F*^2^ > 2σ(*F*^2^)] = 0.068	Hydrogen site location: inferred from neighbouring sites
*wR*(*F*^2^) = 0.159	H atoms treated by a mixture of independent and constrained refinement
*S* = 1.03	*w* = 1/[σ^2^(*F*_o_^2^) + (0.0613*P*)^2^] where *P* = (*F*_o_^2^ + 2*F*_c_^2^)/3
2570 reflections	(Δ/σ)_max_ < 0.001
205 parameters	Δρ_max_ = 0.24 e Å^−^^3^
3 restraints	Δρ_min_ = −0.18 e Å^−^^3^

### Special details

**Table:**

Geometry. All e.s.d.'s (except the e.s.d. in the dihedral angle between two l.s. planes) are estimated using the full covariance matrix. The cell e.s.d.'s are taken into account individually in the estimation of e.s.d.'s in distances, angles and torsion angles; correlations between e.s.d.'s in cell parameters are only used when they are defined by crystal symmetry. An approximate (isotropic) treatment of cell e.s.d.'s is used for estimating e.s.d.'s involving l.s. planes.
Refinement. Refinement of *F*^2^ against ALL reflections. The weighted *R*-factor *wR* and goodness of fit *S* are based on *F*^2^, conventional *R*-factors *R* are based on *F*, with *F* set to zero for negative *F*^2^. The threshold expression of *F*^2^ > σ(*F*^2^) is used only for calculating *R*-factors(gt) *etc*. and is not relevant to the choice of reflections for refinement. *R*-factors based on *F*^2^ are statistically about twice as large as those based on *F*, and *R*- factors based on ALL data will be even larger.

### Fractional atomic coordinates and isotropic or equivalent isotropic displacement parameters (Å^2^)

**Table:**

	*x*	*y*	*z*	*U*_iso_*/*U*_eq_	Occ. (<1)
N1	0.2622 (2)	0.4522 (3)	0.14012 (12)	0.0466 (8)	
N2	0.19869 (19)	0.3787 (3)	0.17652 (11)	0.0447 (7)	
O1	0.43832 (18)	0.5280 (3)	0.10901 (11)	0.0693 (8)	
O2	0.14022 (18)	0.5770 (3)	0.09575 (10)	0.0661 (8)	
O4	0.02926 (17)	−0.0612 (3)	0.37116 (11)	0.0641 (8)	
H4	−0.0227	−0.0192	0.3788	0.096*	
C1	0.4002 (3)	0.6162 (5)	0.06652 (15)	0.0550 (10)	
C2	0.2990 (3)	0.6235 (4)	0.06167 (14)	0.0475 (9)	
C3	0.2609 (3)	0.7113 (5)	0.01798 (16)	0.0683 (12)	
H3A	0.1939	0.7164	0.0137	0.082*	
C4	0.3189 (4)	0.7910 (6)	−0.01924 (18)	0.0890 (15)	
H4A	0.2915	0.8488	−0.0482	0.107*	
C5	0.4165 (4)	0.7845 (6)	−0.0133 (2)	0.0914 (16)	
H5	0.4558	0.8392	−0.0383	0.110*	
C6	0.4583 (3)	0.6986 (5)	0.02896 (18)	0.0740 (13)	
H6	0.5255	0.6955	0.0325	0.089*	
C7	0.5400 (3)	0.5248 (5)	0.11890 (18)	0.0830 (14)	
H7A	0.5725	0.4839	0.0866	0.124*	
H7B	0.5535	0.4577	0.1503	0.124*	
H7C	0.5625	0.6306	0.1265	0.124*	
C8	0.2274 (3)	0.5489 (4)	0.10027 (14)	0.0454 (9)	
C9	0.2381 (3)	0.2932 (4)	0.21392 (14)	0.0467 (9)	
H9	0.3054	0.2881	0.2154	0.056*	
C10	0.1824 (2)	0.2036 (4)	0.25412 (14)	0.0417 (8)	
C11	0.0823 (2)	0.1919 (4)	0.25076 (14)	0.0468 (9)	
H11	0.0500	0.2444	0.2221	0.056*	
C13	0.0768 (3)	0.0289 (4)	0.33218 (14)	0.0446 (9)	
C15	0.2287 (2)	0.1215 (4)	0.29653 (14)	0.0513 (10)	
H15	0.2959	0.1245	0.2991	0.062*	
H1	0.3270 (8)	0.446 (4)	0.1439 (15)	0.080*	
O3	−0.06579 (19)	0.0949 (4)	0.27963 (12)	0.0658 (13)	0.809 (6)
H3B	−0.0941	0.0894	0.3096	0.099*	0.809 (6)
C12	0.0295 (2)	0.1053 (4)	0.28842 (15)	0.0473 (9)	0.809 (6)
C14	0.1761 (3)	0.0351 (4)	0.33496 (15)	0.0515 (10)	0.809 (6)
H14A	0.2083	−0.0195	0.3631	0.062*	0.809 (6)
O3'	0.2166 (10)	−0.0452 (18)	0.3729 (5)	0.085 (6)	0.191 (6)
H3'A	0.1752	−0.0887	0.3921	0.128*	0.191 (6)
C12'	0.1761 (3)	0.0351 (4)	0.33496 (15)	0.0515 (10)	0.191 (6)
C14'	0.0295 (2)	0.1053 (4)	0.28842 (15)	0.0473 (9)	0.191 (6)
H14B	−0.0375	0.0976	0.2848	0.057*	0.191 (6)

### Atomic displacement parameters (Å^2^)

**Table:**

	*U*^11^	*U*^22^	*U*^33^	*U*^12^	*U*^13^	*U*^23^
N1	0.0481 (17)	0.0459 (19)	0.0458 (18)	0.0011 (16)	0.0106 (16)	0.0062 (16)
N2	0.0495 (17)	0.0410 (18)	0.0435 (17)	0.0027 (14)	0.0091 (15)	0.0033 (15)
O1	0.0498 (17)	0.086 (2)	0.0719 (19)	−0.0096 (14)	0.0032 (14)	0.0102 (16)
O2	0.0530 (17)	0.0765 (19)	0.0689 (18)	0.0119 (14)	0.0049 (13)	0.0195 (15)
O4	0.0639 (18)	0.0618 (18)	0.0665 (18)	0.0026 (13)	0.0144 (14)	0.0224 (15)
C1	0.070 (3)	0.051 (2)	0.044 (2)	−0.012 (2)	0.011 (2)	−0.009 (2)
C2	0.063 (2)	0.042 (2)	0.038 (2)	−0.0035 (19)	0.0095 (19)	−0.0021 (18)
C3	0.089 (3)	0.066 (3)	0.051 (3)	0.004 (2)	0.004 (2)	0.009 (2)
C4	0.128 (4)	0.079 (3)	0.060 (3)	−0.003 (3)	0.014 (3)	0.023 (3)
C5	0.135 (5)	0.081 (4)	0.058 (3)	−0.030 (4)	0.033 (3)	0.005 (3)
C6	0.082 (3)	0.078 (3)	0.062 (3)	−0.025 (2)	0.025 (2)	−0.012 (3)
C7	0.059 (3)	0.089 (4)	0.101 (4)	−0.008 (2)	−0.002 (2)	−0.012 (3)
C8	0.060 (3)	0.039 (2)	0.037 (2)	0.0035 (18)	0.0050 (19)	−0.0050 (18)
C9	0.045 (2)	0.046 (2)	0.048 (2)	0.0032 (18)	0.0069 (18)	−0.0037 (19)
C10	0.042 (2)	0.039 (2)	0.044 (2)	0.0030 (17)	0.0043 (18)	−0.0017 (18)
C11	0.054 (2)	0.038 (2)	0.048 (2)	0.0012 (18)	−0.0066 (18)	0.0098 (18)
C13	0.053 (2)	0.035 (2)	0.046 (2)	0.0016 (17)	0.0073 (19)	0.0017 (18)
C15	0.044 (2)	0.062 (3)	0.049 (2)	0.0040 (19)	−0.0021 (18)	0.006 (2)
O3	0.035 (2)	0.078 (3)	0.084 (2)	−0.0011 (16)	0.0014 (15)	0.0331 (18)
C12	0.044 (2)	0.041 (2)	0.058 (2)	0.0004 (17)	−0.0027 (18)	0.0087 (19)
C14	0.055 (2)	0.060 (3)	0.039 (2)	0.011 (2)	−0.005 (2)	0.010 (2)
O3'	0.077 (11)	0.120 (15)	0.059 (11)	−0.005 (9)	0.004 (8)	0.014 (9)
C12'	0.055 (2)	0.060 (3)	0.039 (2)	0.011 (2)	−0.005 (2)	0.010 (2)
C14'	0.044 (2)	0.041 (2)	0.058 (2)	0.0004 (17)	−0.0027 (18)	0.0087 (19)

### Geometric parameters (Å, º)

**Table:**

N1—C8	1.345 (4)	C6—H6	0.9300
N1—N2	1.383 (4)	C7—H7A	0.9600
N1—H1	0.899 (10)	C7—H7B	0.9600
N2—C9	1.273 (4)	C7—H7C	0.9600
O1—C1	1.367 (4)	C9—C10	1.446 (4)
O1—C7	1.423 (4)	C9—H9	0.9300
O2—C8	1.230 (4)	C10—C15	1.386 (4)
O4—C13	1.372 (4)	C10—C11	1.387 (4)
O4—H4	0.8200	C11—C12	1.371 (4)
C1—C6	1.391 (5)	C11—H11	0.9300
C1—C2	1.403 (5)	C13—C14	1.371 (4)
C2—C3	1.386 (5)	C13—C12	1.394 (5)
C2—C8	1.493 (5)	C15—C14	1.381 (4)
C3—C4	1.374 (5)	C15—H15	0.9300
C3—H3A	0.9300	O3—C12	1.334 (4)
C4—C5	1.356 (6)	O3—H3B	0.8200
C4—H4A	0.9300	C14—H14A	0.9300
C5—C6	1.372 (6)	O3'—H3'A	0.8200
C5—H5	0.9300		
			
C8—N1—N2	119.5 (3)	H7A—C7—H7C	109.5
C8—N1—H1	117 (2)	H7B—C7—H7C	109.5
N2—N1—H1	123 (2)	O2—C8—N1	121.9 (3)
C9—N2—N1	115.3 (3)	O2—C8—C2	120.8 (3)
C1—O1—C7	120.9 (3)	N1—C8—C2	117.3 (3)
C13—O4—H4	109.5	N2—C9—C10	122.6 (3)
O1—C1—C6	122.2 (4)	N2—C9—H9	118.7
O1—C1—C2	117.9 (3)	C10—C9—H9	118.7
C6—C1—C2	119.9 (4)	C15—C10—C11	117.8 (3)
C3—C2—C1	117.7 (4)	C15—C10—C9	120.3 (3)
C3—C2—C8	116.2 (3)	C11—C10—C9	121.8 (3)
C1—C2—C8	126.1 (3)	C12—C11—C10	121.9 (3)
C4—C3—C2	122.0 (4)	C12—C11—H11	119.0
C4—C3—H3A	119.0	C10—C11—H11	119.0
C2—C3—H3A	119.0	C14—C13—O4	117.8 (3)
C5—C4—C3	119.4 (5)	C14—C13—C12	119.1 (3)
C5—C4—H4A	120.3	O4—C13—C12	123.0 (3)
C3—C4—H4A	120.3	C14—C15—C10	120.7 (3)
C4—C5—C6	121.1 (5)	C14—C15—H15	119.7
C4—C5—H5	119.4	C10—C15—H15	119.7
C6—C5—H5	119.4	C12—O3—H3B	109.5
C5—C6—C1	119.9 (4)	O3—C12—C11	117.0 (3)
C5—C6—H6	120.0	O3—C12—C13	123.4 (3)
C1—C6—H6	120.0	C11—C12—C13	119.5 (3)
O1—C7—H7A	109.5	C13—C14—C15	120.9 (3)
O1—C7—H7B	109.5	C13—C14—H14A	119.6
H7A—C7—H7B	109.5	C15—C14—H14A	119.6
O1—C7—H7C	109.5		

### Hydrogen-bond geometry (Å, º)

**Table:**

*D*—H···*A*	*D*—H	H···*A*	*D*···*A*	*D*—H···*A*
O4—H4···O2^i^	0.82	1.91	2.730 (3)	174
O3—H3*B*···N2^i^	0.82	2.31	2.789 (4)	118
O3—H3*B*···O2^i^	0.82	2.36	3.166 (4)	167
N1—H1···O1	0.90 (1)	1.88 (3)	2.620 (4)	138 (3)

Symmetry code: (i) −*x*, *y*−1/2, −*z*+1/2.
